# Advancing Evidence-Based Medicine for Population, Intervention, Comparison, and Outcome Element Recognition and Extraction in Medical Literature: Large Language Model Approach

**DOI:** 10.2196/91215

**Published:** 2026-08-14

**Authors:** Zeyuan Hao, Yifan Duan, Yu Wang

**Affiliations:** 1 School of Software Engineering, Beijing Jiaotong University, 3 Shangyuan Village, Haidian District, Beijing, 100044, China, 86 18404966218; 2 Institute of Medical Information/Medical Library, Chinese Academy of Medical Sciences & Peking Union Medical College, Beijing, China; 3 College of Computer and Information Engineering, Nanjing Tech University, Nanjing, China

**Keywords:** evidence-based medicine, population, intervention, comparison, outcome, PICO, large language model, LLM, prompt engineering, parameter-efficient fine-tuning, natural language processing

## Abstract

**Background:**

The exponential expansion of biomedical literature has created an urgent need for efficient methods to recognize and extract population, intervention, comparison, and outcome (PICO) elements—the foundational elements of evidence-based medicine.

**Objective:**

This study systematically evaluated 2 complementary approaches for automating PICO recognition and extraction in medical literature: prompt engineering optimization and parameter-efficient fine-tuning (PEFT) of large language models (LLMs).

**Methods:**

We developed a dual-phase methodological framework: (1) systematic prompt optimization incorporating in-context learning, chain of thought (COT), and multipath reasoning strategies; and (2) PEFT of the LLM architecture using low-rank adaptation (LoRA), quantized LoRA, and freeze techniques. The PubMed-PICO and NICTA-PIBOSO benchmark datasets were used for recognition tasks, and the EBM-NLP dataset was used for extraction tasks. Performance metrics included precision, recall, and *F*_1_-score. *F*_1_-score was adopted as the major metric as it balances precision and recall.

**Results:**

For prompt engineering, COT achieved the overall best performance across both recognition and extraction tasks. For example, in the recognition task, COT obtained strong average *F*_1_-scores of 77.1% (SD 0.5%) for the population element and 84.5% (SD 0.4%) for the outcome element on PubMed-PICO. In the extraction task, COT achieved the highest average *F*_1_-score of 73.9% across 3 PICO elements (the population, intervention, and outcome elements) on EBM-NLP. These results suggest that, for smaller models such as those with 3B parameters, explicit step-by-step guidance in COT is more effective than more complex prompting strategies. In PEFT implementations, for example, LoRA achieved the best recognition performance (mean *F*_1_-score 91.7%, SD 0.3% for population) on PubMed-PICO, whereas quantized LoRA showed the best extraction capability (mean *F*_1_-score 79.3%, SD 0.5% for intervention) on EBM-NLP. Fine-tuned models achieved competitive performance across all datasets, with notable gains on NICTA-PIBOSO and EBM-NLP. PEFT further enhanced the model’s overall performance compared with prompt engineering, with element-dependent differences across PICO categories.

**Conclusions:**

Our findings indicate that LLMs can effectively automate PICO recognition and extraction through 2 complementary approaches. First, prompt engineering allows the model to perform tasks directly without altering its internal settings. Second, the PEFT method further unlocks their maximum performance potential by incorporating additional fine-tuning based on prompt engineering. This work makes significant advances and provides critical insights for optimizing methodological approaches in clinical applications related to or comprising PICO extraction and recognition tasks.

## Introduction

### Background

Evidence-based medicine (EBM) represents a paradigm shift in clinical practice, fundamentally anchored in “the conscientious, explicit, and judicious use of current best evidence in making decisions about the care of individual patients” [1]. This approach integrates clinical research with practical expertise to optimize therapeutic decision-making. Central to EBM implementation are the population, intervention, comparison, and outcome (PICO) elements—which serve as the cornerstone for formulating precise clinical questions and evaluating medical evidence. However, the exponential growth of biomedical literature poses significant challenges in efficiently extracting these critical elements from vast textual repositories.

Recent advancements in large language models (LLMs) have revolutionized natural language processing capabilities, demonstrating exceptional performance across diverse biomedical applications, including named entity recognition (NER) [2,3], knowledge graph construction [4,5], intelligent agent development [6,7], and question-answering systems [8,9]. The emergence of open-source LLM architectures (eg, Llama, Qwen, and DeepSeek) has further catalyzed domain-specific models, exemplified by specialized models such as PMC-Llama [10] and BioMedGPT-LM [11]. These developments show that LLMs can be used for recognition and extraction tasks in medical fields. Considering graphics processing unit (GPU) resource availability and deployment efficiency without compromising the generalization of the study, we selected the Llama 3.2-3B model (Meta AI), which has a small parameter size yet high model performance, to develop a two-stage PICO element recognition and extraction method: (1) validate the reasoning capability of prompt engineering based on the base model and (2) implement parameter-efficient fine-tuning (PEFT) based on Llama 3.2-3B. Our work makes two primary contributions:

1. Designing and evaluating modular prompt templates and frameworks for PICO element recognition and extraction
2. Developing a fine-tuned LLM optimized for PICO element recognition and extraction and providing a comprehensive comparative analysis of 3 predominant PEFT techniques (low-rank adaptation [LoRA], freeze, and quantized LoRA [QLoRA])

### Related Work

In this study, “PICO recognition task" refers to assigning text segments to predefined PICO categories, which is a classification problem, and “PICO extraction task” refers to identifying the specific spans of PICO elements in text, which is an NER problem. Current mainstream approaches primarily encompass rule-based or dictionary-based methods, machine learning techniques, deep learning architectures, and LLM-based methodologies. This section reviews relevant research by analyzing the trajectory of technological evolution.

One stream is to use predefined matching rules or domain-specific lexicons for entity recognition. Demner-Fushman and Lin [12] pioneered rule-based pattern matching in 2007 for automated PICO element extraction from structured abstracts. Cohen et al [13] enhanced literature screening efficiency by integrating rule-based systems with basic statistical methods to identify PICO-containing articles. While these methods achieve high accuracy, they suffer from poor generalization capabilities and significant maintenance challenges for rule or dictionary updates.

Conventional machine learning implementations include support vector machines, decision trees, hidden Markov models, and conditional random fields. Notable applications include the naive Bayes classifier by Huang et al [14] for PICO identification in structured abstracts, the conditional random fields–based framework with feature templates for EBM text analysis by Hassanzadeh et al [15], and the n-gram–based ExaCT system for extracting population and intervention elements from clinical trial summaries by Kiritchenko et al [16]. Boudin et al [17] conducted comprehensive comparative analyses of multiple machine learning models. Although superior to rule-based approaches, these methods remain constrained by their reliance on expert-crafted feature engineering.

Deep neural networks have substantially reduced feature engineering burdens while improving performance. Predominant architectures include bidirectional long short-term memory, convolutional neural networks, and deep neural networks. Nye et al [18] developed an attention-enhanced sequence-labeling model validated on PICO annotation datasets. Wang et al [19] implemented bidirectional encoder representations from transformers–based PICO sequence labeling with multitask learning optimization. Stylianou et al [20] proposed an end-to-end PICO recognition system using recurrent neural networks. While they outperform traditional machine learning methods, deep learning approaches require substantial annotated training data.

The emergence of LLMs has catalyzed novel recognition methodologies requiring minimal domain-specific annotations through fine-tuning. These models have demonstrated notable successes in biomedical NER tasks [2,21-23]. However, research on PICO recognition and extraction using LLMs remains relatively scarce. This study focused on investigating LLM capabilities for this specialized task.

## Methods

### Overall Framework

**Figure 1.**
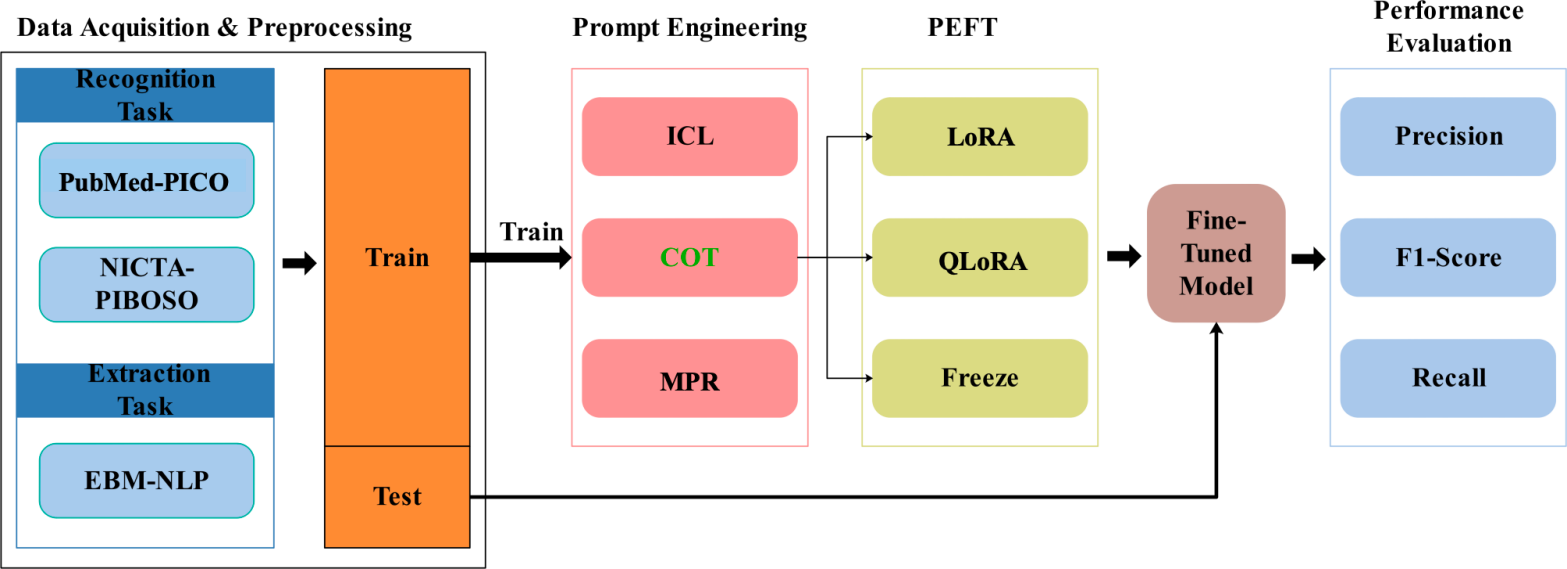
Research framework. COT: chain of thought; ICL: in-context learning; LoRA: low-rank adaptation; MPR: multi-path reasoning; PEFT: parameter-efficient fine-tuning; QLoRA: quantized LoRA.

As depicted in Figure 1, this study explored the feasibility and effectiveness of LLMs for recognizing and extracting PICO elements in EBM literature through a 4-step workflow.

First, 3 EBM corpora—the PubMed-PICO and NICTA-PIBOSO datasets for the recognition task and the EBM-NLP dataset for the extraction task—were systematically collected, preprocessed, and divided into training and testing sets.

Second, various prompt engineering strategies were designed, including in-context learning (ICL), chain of thought (COT), and multi-path reasoning (MPR), to tailor the prompts for each task, and comparative experiments were conducted to evaluate the effectiveness of these strategies.

Third, building on the results of the second stage and using the best-performing prompt, a comparative study using PEFT was carried out. In this phase, methods such as LoRA, QLoRA, and freeze were applied to refine the model further, thereby providing deeper insights into the impact of different fine-tuning algorithms and ultimately yielding a robust, fine-tuned LLM.

Finally, the model’s performance was evaluated using standard confusion matrix metrics (precision, *F*_1_-score, and recall), thereby quantifying its effectiveness in accurately identifying PICO elements.

### Datasets

This study investigated the performance of LLMs in PICO element recognition and extraction tasks. To achieve this objective, we used 3 datasets with the following configurations. For the recognition task, we used 2 high-quality annotated open-source datasets: PubMed-PICO [24] and NICTA-PIBOSO [25]. The PubMed-PICO dataset, developed in 2018, originally classifies each sentence into 7 categories: aim, population, intervention, outcome, method, results, and comparisons. As comparisons are often inconsistently reported and semantically overlap with intervention descriptions (eg, placebo, standard care, or other interventions), we excluded this category to standardize task settings across datasets. In addition, following related dataset conventions and prior studies on NICTA-PIBOSO [25,26], and aligned with our research objectives, we focused on the population, intervention, and outcome triadic recognition framework. The NICTA-PIBOSO dataset originally classifies each sentence into 6 categories: population, intervention, background, outcome, study design, and other. Following the aforementioned standardization principle, we likewise restricted our analysis to population, intervention, and outcome annotations in this dataset. The population, intervention, and outcome distribution characteristics of both datasets are summarized in Table 1.

**Table 1.** Data statistics of the NICTA-PIBOSO and PubMed-PICO datasets.

	NICTA-PIBOSO sentences, n	PubMed-PICO sentences, n
Population	812	25,070
Intervention	690	22,195
Outcome	4523	29,448

For the extraction task, we used the EBM-NLP dataset [18], which differs from the aforementioned 2 recognition datasets in that it is specifically designed for NER tasks. This corpus adopts the begin-inside-outside tagging scheme to annotate PICO elements at the token level within abstract sentences, thereby satisfying formal requirements for entity boundary identification. Notably, individual sentences in this dataset frequently contain multiple entity types—a characteristic that necessitated strategic preprocessing to optimize LLM performance. Guided by the classic computer science principle of “divide and conquer,” we designed a prompt mechanism that allows multiple inferences to be made on the same text, each targeting only one category while preserving the original context. Finally, the model aggregates these different category extraction results and outputs them in JSON format.

For all 3 datasets, we adopted the official train-test splits provided by the original benchmark settings.

### Design of the Prompt

#### Overview

In the context of LLMs, the NER task has been innovatively reformulated as a question-answering paradigm. This approach transforms unstructured text inputs into standardized query formats through prompt engineering. LLMs then generate structured outputs. The optimization of prompt engineering emerges as a critical determinant that demonstrates a direct correlation with enhanced performance metrics.

Following established prompt composition principles [27], our methodology implemented 3 key strategies. First, we established explicit instruction specifications with contextual constraints to eliminate semantic ambiguities. Second, we used hierarchical task decomposition to process complex queries through multistage response protocols, thereby reducing cognitive load in LLM processing. Finally, we incorporated domain-specific ontological terminology into the prompts to anchor model responses within relevant knowledge boundaries, thereby improving the consistency and faithfulness of entity extraction.

This PICO element recognition and extraction study compared the performance of 3 prompting strategies: ICL, COT, and MPR. Our methodological framework incorporates specifically engineered prompt templates developed through rigorous experimental design. The architecture implementation was conducted in the key phases described below.

#### Baseline Prompt Construction

We established dual baseline prompt templates for recognition and extraction tasks, serving as foundational frameworks for subsequent ICL, COT, and MPR. Each template integrates four core components (delimited by “$$,” which is used solely as a separator between the four core components of the prompt template and has no mathematical meaning):

1. Task definition—activates LLM understanding of target objectives
2. Operational instructions—specify analytical requirements using chromatic text coding (green: PICO element identification; red: critical precautions; orange: output formatting constraints)
3. Input schema—define data structure requirements (highlighted in pink)
4. Output specifications—prescribe response organization protocols

The extraction task template implements a divide-and-conquer strategy through entity-specific prompting mechanisms. This hierarchical approach enables targeted optimization for individual PICO components while maintaining systemic coherence.

**Figure 2.**
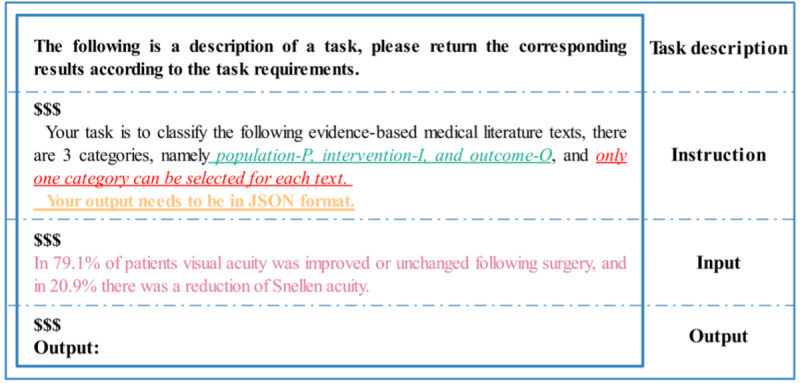
Baseline prompt template for the recognition task.

**Figure 3.**
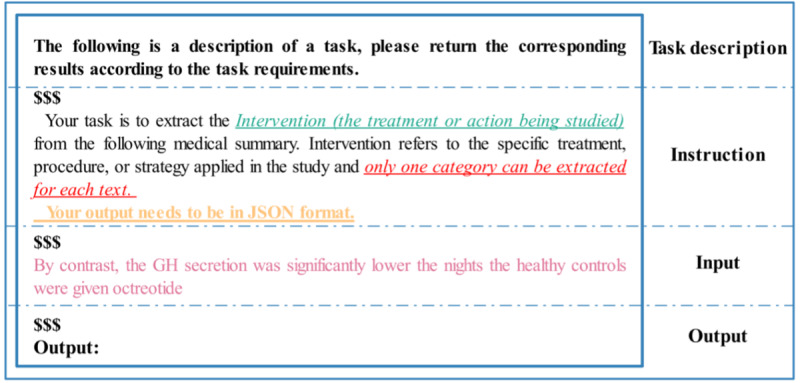
Baseline prompt template for the extraction task.

Figures 2 and 3 illustrate the baseline prompt templates for the 2 tasks.

#### Integration of the ICL Strategy Into Prompt Templates

**Figure 4.**
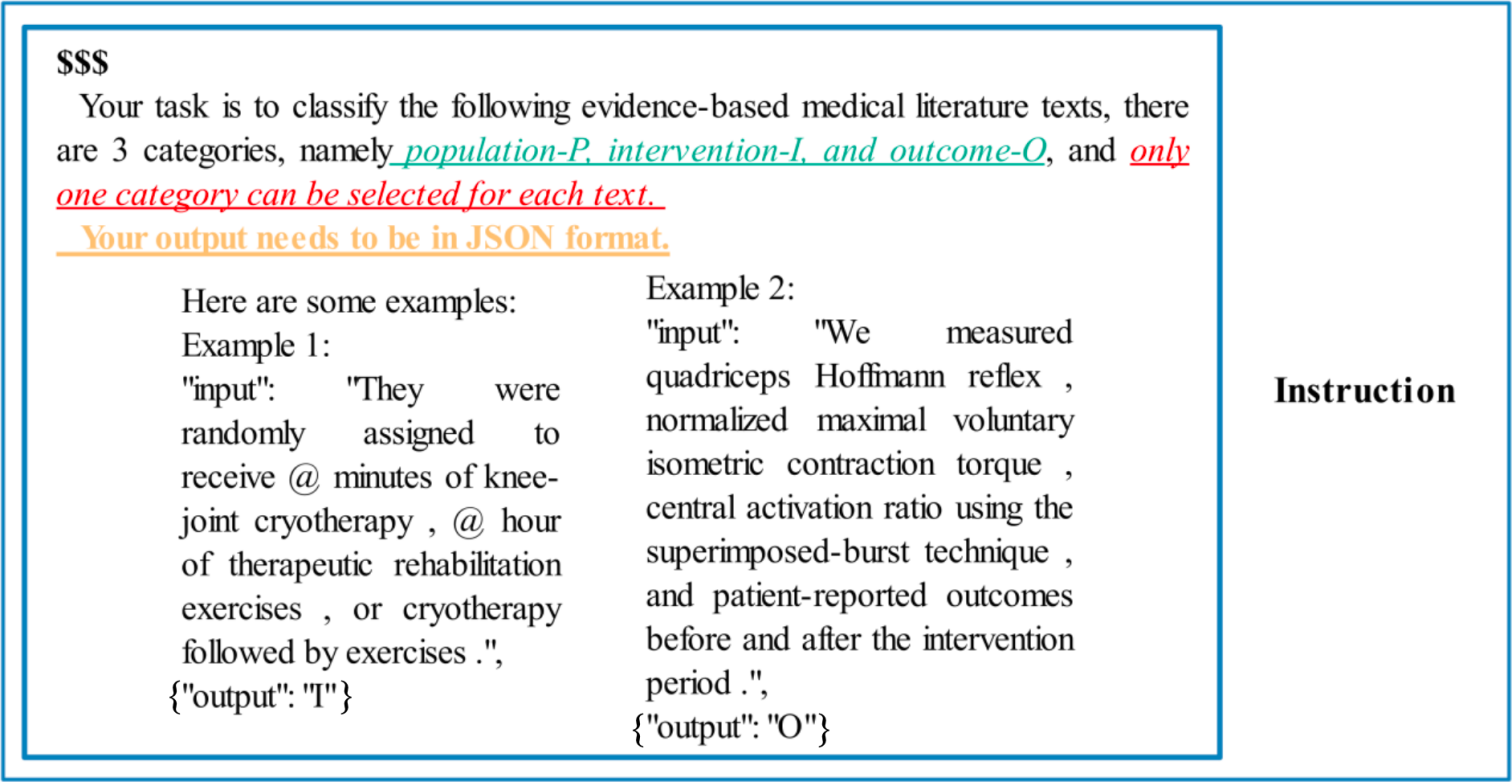
In-context learning prompt template for the recognition task (instruction part).

**Figure 5.**
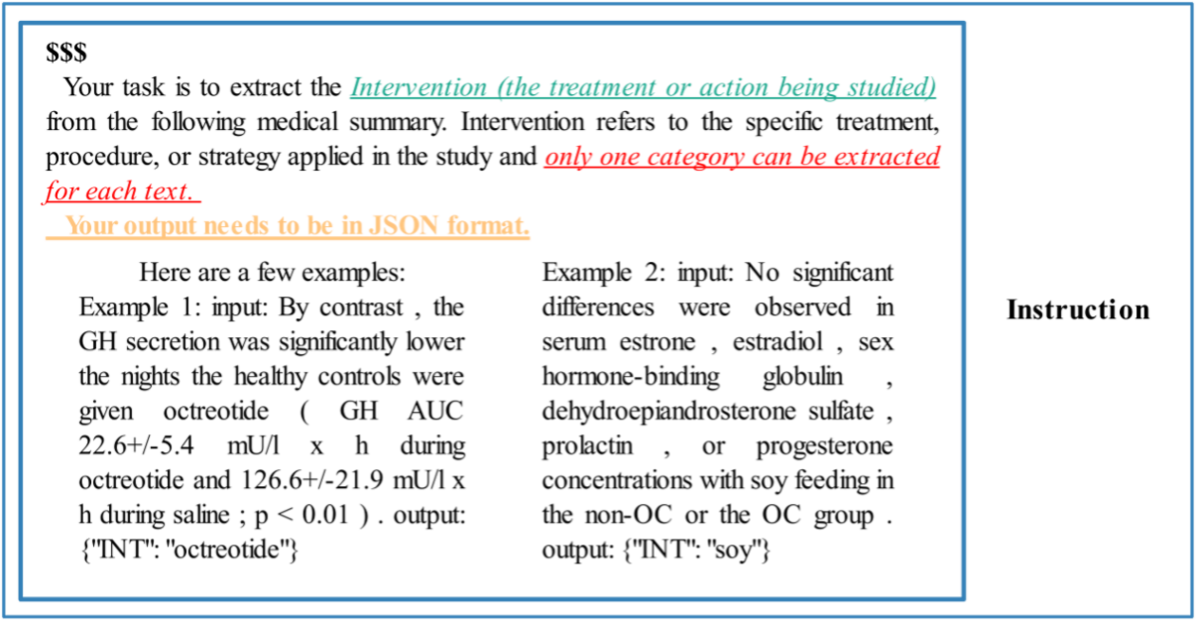
In-context learning prompt template for the extraction task (instruction part).

The instruction section of the baseline prompt template was augmented with contextually relevant prompt descriptors to implement the ICL strategy. For ICL, 2 in-context examples were fixed in the prompt template before inference and reused across all test instances. The same examples were applied consistently throughout inference. The task-specific instructional schemata for both experimental paradigms are illustrated in Figures 4 and 5.

#### Development of COT and MPR Prompt Architectures

**Figure 6.**
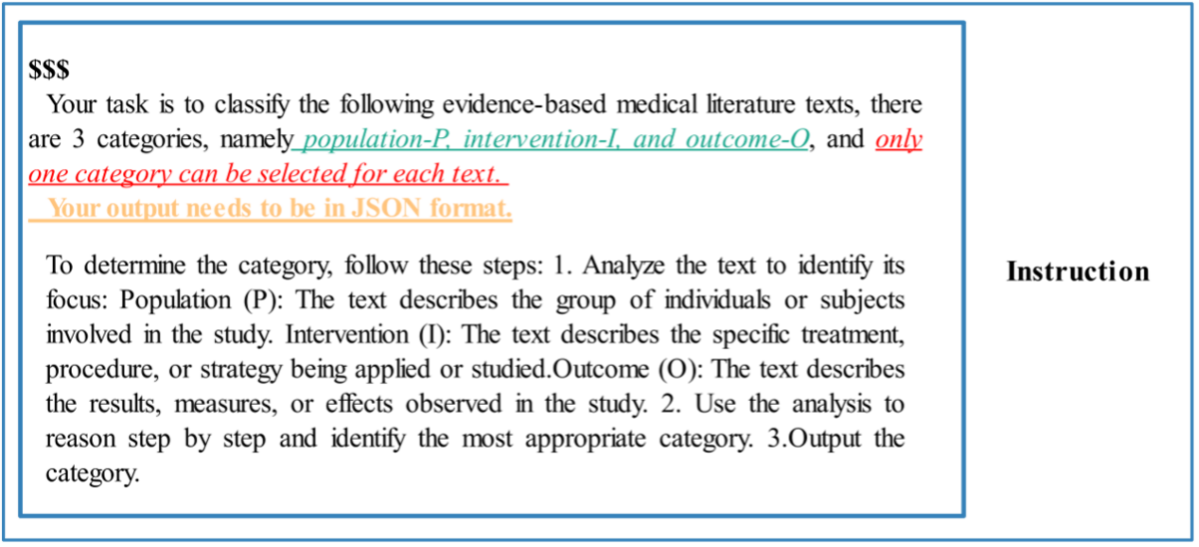
Chain-of-thought prompt template for the recognition task (instruction part).

**Figure 7.**
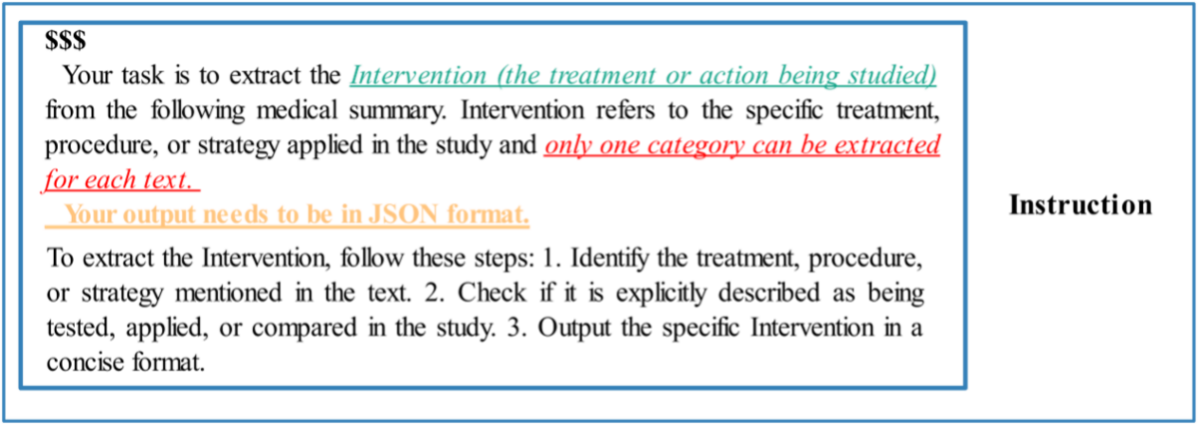
Chain-of-thought prompt template for the extraction task (instruction part).

**Figure 8.**
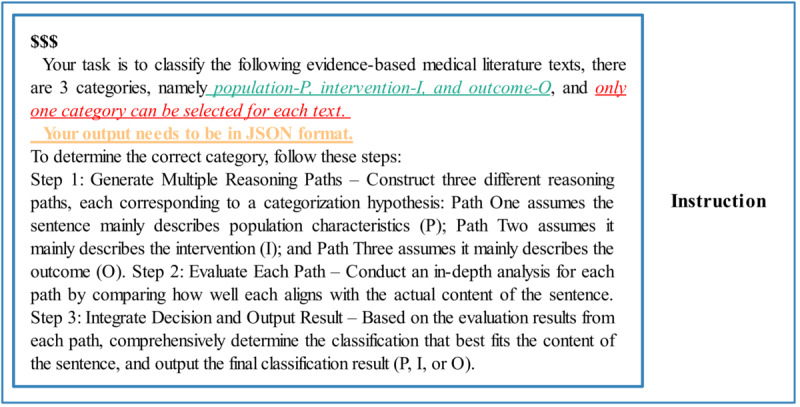
Multi-path reasoning prompt template for the recognition task (instruction part).

**Figure 9.**
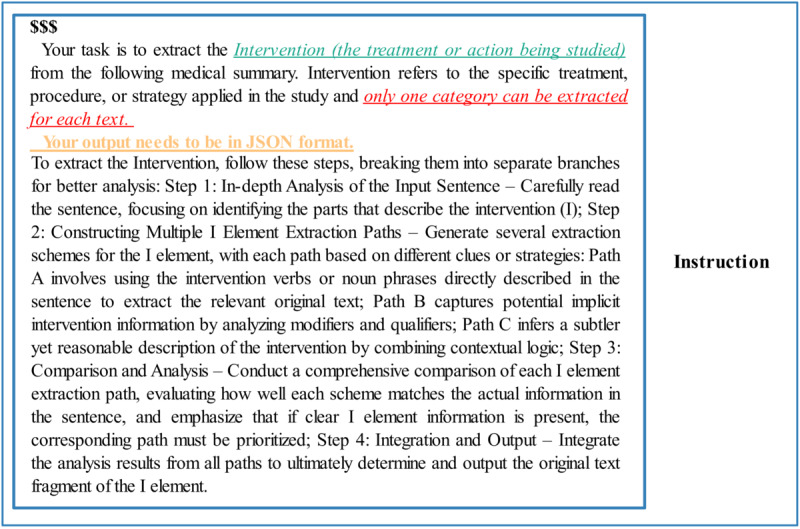
Multi-path reasoning prompt template for extraction task (instruction part).

Guided by the methodological frameworks established in COT [28] and MPR [29] prompting paradigms, we developed corresponding reasoning-enhanced templates for both the recognition and extraction tasks. For the COT and MPR settings, the reasoning procedure was specified in the prompt instructions, and text generation was controlled via shared decoding settings across prompt-based experiments (temperature=0.5; max_new_tokens=1000; repetition_penalty=1.2; do_sample=True). The operational specifics of these cognitive scaffolding mechanisms are schematically delineated in Figures 6-9.

### LLM Fine-Tuning

In addition to conducting comparisons of various prompt engineering strategies across different tasks, our investigation extended to an empirical evaluation of fine-tuning methodologies. Given the computational intensiveness inherent in full-parameter fine-tuning, this constraint has catalyzed the emergence of numerous PEFT algorithms. These approaches maintain model performance while requiring adjustment of only minimal parameter subsets.

Through an investigation of the compatibility between various PEFT algorithms, this study selected the following PEFT methods:

- LoRA [30]—introduces trainable low-rank matrices (A and B) next to the original weight matrix through low-rank decomposition, only updating the parameters of these low-rank subspaces while freezing the original model weights
- QLoRA [31]—enhances LoRA through 4-bit quantization and paged optimization techniques, achieving 70% reduction in GPU memory consumption while maintaining model accuracy
- Freeze [32]—freezes most of the base model parameters and fine-tunes only the final 2 transformer layers

### Experimental Setup and Evaluation Metrics

All model training procedures throughout this experimental framework were conducted on an NVIDIA GeForce RTX 4090 D 24-GB GPU. For PEFT experiments, the per-device batch size was 8, with gradient accumulation steps of 1, resulting in an effective batch size of 8. All models were trained for 2 epochs using the AdamW optimizer (β1=0.9; β2=0.999; ε=1 × 10^–8^), a cosine learning rate scheduler, and a warm-up ratio of 0.1. Early stopping was not used. The learning rate was 5 × 10^–5^ for freeze, LoRA, and QLoRA. The validation split ratio was 0.1. For LoRA and QLoRA, the LoRA rank and α were both set to 8, with a LoRA dropout of 0.05, and the target modules were q_proj and v_proj. For QLoRA, 4-bit quantization with NF4, double quantization, and BF16 compute data type were used. For freeze, we trained the last 2 layers. The compute data type was BF16 for all PEFT settings.

For performance assessment, we adopted well-established confusion matrix–derived evaluation metrics, with particular focus on 3 critical indexes: precision, recall, and *F*_1_-score. We used macro–*F*_1_-score (the unweighted mean of the class-specific *F*_1_-score for population, intervention, and outcome) as it provides a balanced overall assessment of precision and recall while reducing bias toward majority classes. Precision and recall were also reported separately as false negatives and false positives may have different practical implications in EBM applications. For the extraction task on the EBM-NLP dataset, model outputs were parsed from JSON format and evaluated using strict exact matches. A prediction was counted as correct only when the extracted mention text exactly matched the gold-standard mention text for the corresponding entity type; malformed or incomplete outputs were treated as errors. These metrics were defined as follows:

Precision = TP/(TP + FP) (1)

Recall = TP/(TP + FN) (2)

*F*_1_-score = (2 × TP)/(2 × TP + FP +FN) (3)

In these equations, “TP,” “FP,” and “FN” denote true positives, false positives, and false negatives, respectively. To assess result stability, all experiments were repeated 3 times, and the results are reported as means and SDs. Statistical analysis for pairwise method comparisons was performed using instance-level bootstrap analysis based on test set predictions, with *F*_1_-score differences, 95% CIs, and approximate 2-sided *P* values reported. Detailed statistical test results are reported in Table S1 in Multimedia Appendix 1.

### Ethical Considerations

This study was a secondary analysis of publicly available datasets and did not involve new human participant recruitment or direct interaction. No identifiable personal information or images were involved. Therefore, additional informed consent and participant compensation were not applicable to this study. Details of the original data collection procedures are available in the original dataset publications.

## Results

This study addressed two primary scientific inquiries: (1) the feasibility of LLMs in performing PICO element identification and extraction tasks and (2) the effectiveness of different prompt engineering and fine-tuning mechanisms for these applications. To systematically investigate these questions, we designed dual experimental frameworks evaluating both prompt engineering strategies and fine-tuning approaches across PICO recognition and extraction tasks.

For the recognition task, Tables 2 and 3 summarize the performance metrics of various prompt engineering methods on the PubMed-PICO and NICTA-PIBOSO datasets. Specifically, on the PubMed-PICO dataset, COT achieved average *F*_1_-scores of 77.1% (SD 0.5%) for population, 70.4% (SD 0.6%) for intervention, and 84.5% (SD 0.4%) for outcome. On the NICTA-PIBOSO dataset, the *F*_1_-scores were 48.3% (SD 1.1%) for population, 46.4% (SD 1.2%) for intervention and 84.7% (SD 0.8%) for outcome. Overall, across both datasets, the COT prompting strategy achieved a stronger average performance than the ICL and MPR approaches. This advantage was further supported by instance-level bootstrap analysis based on test set predictions indicating that COT achieved the most stable and robust overall performance.

**Table 2.** Recognition performance of various prompt engineering methods on the PubMed-PICO dataset (sentence-level classification).

Prompt engineering method	Population element (%), mean (SD)			Intervention element (%), mean (SD)			Outcome element (%), mean (SD)		
	Precision	Recall	*F*_1_-score	Precision	Recall	*F*_1_-score	Precision	Recall	*F*_1_-score
ICL^a^	51.1 (0.7)	93.3 (0.5)	66.1 (0.6)^b^	*85.2 (0.8)* ^c^	14.3 (0.9)	24.5 (0.8)^b^	81.9 (0.6)	73.5 (0.7)	77.5 (0.6)^b^
COT^d^	*74.4 (0.5)*	80.1 (0.6)	77.1 (0.5)^e,f^	80.1 (0.7)	*62.8 (0.8)*	70.4 (0.6)^e,f^	81.5 (0.5)	*87.9 (0.5)*	84.5 (0.4)^e,f^
MPR^g^	38.1 (0.9)	*97.3 (0.4)*	54.8 (0.7)	77.0 (0.8)	12.5 (1.0)	21.5 (0.9)	*91.8 (0.6)*	26.9 (0.8)	41.6 (0.7)

a ICL: in-context learning.

b ICL vs multi-path reasoning.

c Italics indicate the best performance of each indicator.

d COT: chain of thought.

e COT vs ICL.

f COT vs multi-path reasoning.

g MPR: multi-path reasoning.

**Table 3.** Recognition performance of various prompt engineering methods on the NICTA-PIBOSO dataset (sentence-level classification).

Prompt engineering method	Population element (%), mean (SD)			Intervention element (%), mean (SD)			Outcome element (%), mean (SD)		
	Precision	Recall	*F*_1_-score	Precision	Recall	*F*_1_-score	Precision	Recall	*F*_1_-score
ICL^a^	20.3 (1.3)	92.7 (1.0)	33.3 (1.2)	*71.4 (1.5)* ^b^	4.0 (0.8)	7.6 (0.9)	*94.7 (0.8)*	53.5 (1.4)	68.4 (1.1)^c^
COT^d^	34.4 (1.2)	81.3 (1.3)	48.3 (1.1)^e^	53.7 (1.4)	*40.8 (1.5)*	*46.4 (1.2)* ^e,f^	94.6 (0.7)	76.8 (1.0)	*84.7 (0.8)* ^e,f^
MPR^g^	*37.8 (1.3)*	*97.7 (0.6)*	*54.6 (1.1)* ^c^ ^,^ ^f^	76.9 (1.2)	11.6 (1.0)	20.2 (1.0)^c^	92.5 (0.9)	*25.9 (1.3)*	40.5 (1.1)

a ICL: in-context learning.

b Italics indicate the best performance of each indicator.

c ICL vs multi-path reasoning.

d COT: chain of thought.

e COT vs ICL.

f COT vs multi-path reasoning.

g MPR: multi-path reasoning.

We implemented PEFT on the Llama 3.2-3B foundation model, conducting comparative analyses of 3 prominent PEFT methodologies: LoRA, QLoRA, and freeze. The empirical evidence presented in Tables 4 and 5 reveals several critical findings.

**Table 4.** Recognition performance of various parameter-efficient fine-tuning (PEFT) methods on the PubMed-PICO dataset (sentence-level classification).

PEFT method	Population element (%), mean (SD)			Intervention element (%), mean (SD)			Outcome element (%), mean (SD)		
	Precision	Recall	*F*_1_-score	Precision	Recall	*F*_1_-score	Precision	Recall	*F*_1_-score
LoRA^a^	*94.2 (0.3)* ^b^	89.3 (0.4)	*91.7 (0.3)* ^c,d^	88.2 (0.4)	*87.2 (0.4)*	*87.7 (0.3)* ^c,d^	*90.5 (0.3)*	*95.2 (0.2)*	*92.8 (0.2)* ^c,d^
QLoRA^e^	92.8 (0.4)	*90.5 (0.3)*	91.6 (0.3)^f^	*90.0 (0.3)*	85.6 (0.4)	*87.7 (0.3)* ^f^	90.2 (0.3)	95.1 (0.2)	92.7 (0.2)^f^
Freeze	93.9 (0.4)	88.9 (0.4)	91.4 (0.3)	88.3 (0.4)	84.9 (0.5)	86.6 (0.4)	88.9 (0.3)	95.1 (0.3)	92.0 (0.3)

a LoRA: low-rank adaptation.

b Italics indicate the best performance of each indicator.

c LoRA vs quantized LoRA.

d LoRA vs freeze.

e QLoRA: quantized LoRA.

f QLoRA vs freeze.

**Table 5.** Recognition performance of various parameter-efficient fine-tuning (PEFT) methods on the NICTA-PIBOSO dataset (sentence-level classification).

PEFT method	Population element (%), mean (SD)			Intervention element (%), mean (SD)			Outcome element (%), mean (SD)		
	Precision	Recall	*F*_1_-score	Precision	Recall	*F*_1_-score	Precision	Recall	*F*_1_-score
LoRA^a^	*79.2 (0.7)* ^b^	68.7 (0.9)	*72.5 (0.7)* ^c,d^	*78.5 (0.8)*	67.2 (0.9)	72.4 (0.7)^d^	95.2 (0.3)	*98.1 (0.2)*	*96.6 (0.2)* ^c,d^
QLoRA^e^	72.3 (0.9)	*71.6 (0.8)*	71.9 (0.7)^f^	77.9 (0.8)	*70.4 (0.8)*	*74.0 (0.7)* ^c,f^	*95.7 (0.3)*	97.1 (0.3)	96.4 (0.2)^f^
Freeze	78.8 (0.8)	61.7 (1.0)	69.2 (0.8)	68.3 (0.9)	68.8 (0.9)	68.5 (0.8)	94.9 (0.4)	97.1 (0.3)	96.0 (0.3)

a LoRA: low-rank adaptation.

b Italics indicate the best performance of each indicator.

c LoRA vs quantized LoRA.

d LoRA vs freeze.

e QLoRA: quantized LoRA.

f QLoRA vs freeze.

The performance disparity between datasets is worth particular attention. Our analysis revealed a potential correlation with training data characteristics: the PubMed-PICO dataset contains 76,713 entries with balanced element distribution (Table 1), whereas NICTA-PIBOSO has only 6025 samples with skewed distributions (4523, 812, and 690 for the outcome, population, and intervention elements, respectively). This substantial difference in both total sample size and sample unbalanced distribution likely contributed to the superior performance on PubMed-PICO across all evaluation metrics.

For the PubMed-PICO dataset, COT showed a good balance and stable performance between precision and recall. On the other hand, ICL and MPR inconsistently diverged between precision and recall.

Given COT’s demonstrated efficacy, consistency, and balance in preliminary experiments, we adopted COT as the baseline template for subsequent fine-tuning experiments. This methodological continuity ensured comparability between prompt engineering and fine-tuning approaches in our phased investigation.

Our quantitative analysis further revealed that fine-tuning substantially outperformed prompt engineering across all evaluation metrics. For instance, on the PubMed-PICO dataset, the LoRA–fine-tuned model demonstrated substantial improvements: the COT method achieved an average *F*_1_-score of 77.1% (SD 0.5%) for population element recognition. With COT as the chosen prompt engineering strategy, LoRA further enhanced the model, reaching an average *F*_1_-score of 91.7% (SD 0.3%; +14.6%). Similar enhancements were observed in intervention element recognition (+17.3%) and outcome element recognition (+8.3%). This performance gap became more pronounced on the NICTA-PIBOSO dataset, where the LoRA-optimized model outperformed COT prompting by 24.2% in population element recognition. It is noteworthy that the intervention element performance leaped from an average of 46.4% (SD 1.2%) to 72.4% (SD 0.7%), representing a 26% improvement, accompanied by an 11.9% outcome element improvement. Overall, LoRA demonstrated superior performance across both benchmark datasets. Statistical analysis further supported the robustness of this advantage.

Following the 2-phase experimental design, we further conducted PICO element extraction tasks on the EBM-NLP dataset. Initial experiments compared 3 prompting strategies, followed by PEFT experiments with LLMs. The experimental results are presented in Tables 6 and 7.

**Table 6.** Extraction performance of various prompt engineering methods on the EBM-NLP dataset (token-level named entity recognition).

Prompt engineering method	Population element (%), mean (SD)			Intervention element (%), mean (SD)			Outcome element (%), mean (SD)		
	Precision	Recall	*F*_1_-score	Precision	Recall	*F*_1_-score	Precision	Recall	*F*_1_-score
ICL^a^	75.2 (1.0)	*77.1 (1.1)* ^b^	76.1 (0.9)^c^	78.4 (1.0)	*78.3 (1.1)*	*78.3 (0.9)* ^c^ ^,^ ^d^	63.6 (1.3)	66.8 (1.2)	65.2 (1.1)
COT^e^	*88.6 (0.8)*	75.1 (1.1)	*81.3 (0.8)* ^d,f^	*82.7 (0.9)*	68.9 (1.2)	75.2 (0.9)^f^	65.6 (1.1)	65.0 (1.2)	65.3 (1.0)^d^
MPR^g^	61.5 (1.4)	51.8 (1.5)	56.2 (1.2)	60.5 (1.3)	50.5 (1.4)	55.0 (1.2)	*78.1 (0.9)*	*73.2 (1.0)*	*75.6 (0.8)* ^c^ ^,^ ^f^

a ICL: in-context learning.

b Italics indicate the best performance of each indicator.

c ICL vs multi-path reasoning.

d Chain of thought vs ICL.

e COT: chain of thought.

f COT vs multi-path reasoning.

g MPR: multi-path reasoning.

**Table 7.** Extraction performance of various parameter-efficient fine-tuning (PEFT) methods on the EBM-NLP dataset (token-level named entity recognition)^a^.

PEFT method	Population element (%), mean (SD)			Intervention element (%), mean (SD)			Outcome element (%), mean (SD)		
	Precision	Recall	*F*_1_-score	Precision	Recall	*F*_1_-score	Precision	Recall	*F*_1_-score
LoRA^b^	81.6 (0.5)	76.7 (0.6)	79.1 (0.5)	86.7 (0.5)	72.1 (0.6)	78.7 (0.5)^c^	80.2 (0.6)	67.2 (0.7)	73.1 (0.6)^c^
QLoRA^d^	*82.0 (0.5)* ^e^	*76.9 (0.5)*	*79.3 (0.5)* ^f,g^	*87.3 (0.5)*	*72.6 (0.6)*	*79.3 (0.5)* ^f,g^	*81.1 (0.5)*	*68.0 (0.6)*	*74.0 (0.5)* ^f,g^
Freeze	81.7 (0.5)	76.8 (0.6)	79.2 (0.5)^c^	83.2 (0.7)	69.1 (0.8)	75.5 (0.6)	78.0 (0.6)	65.3 (0.7)	71.1 (0.6)

a We compared our experimental results with those reported in previous studies using the same 3 datasets. Jin and Szolovits [24] developed the CPED-BioBERT model based on deep neural networks through extensive work on PubMed-PICO and NICTA-PIBOSO. Their model achieved *F*_1_-scores of 91.0% for population elements, 84.6% for intervention elements, and 88.9% for outcome elements on the PubMed-PICO dataset, which were consistently lower than those achieved by our low-rank adaptation–fine-tuned model.

b LoRA: low-rank adaptation.

c LoRA vs freeze.

d QLoRA: quantized LoRA.

e Italics indicate the best performance of each indicator.

f LoRA vs QLoRA.

g QLoRA vs freeze.

In the prompt-based extraction experiments, consistent with findings from the recognition task, the COT method achieved an average *F*_1_-score of 73.9% across all 3 elements, surpassing ICL (73.2%) and MPR (62.3%). It also showed superior average precision (79.0% vs 72.4% for ICL and 66.7% for MPR). These results collectively indicate the strongest average performance of COT prompting. Overall, COT achieved the highest overall average *F*_1_-score. This advantage was further supported by statistical analysis.

For the fine-tuning experiments, we compared 3 algorithms (LoRA, QLoRA, and freeze) in LLM-based information extraction. In contrast to findings from the recognition task, QLoRA demonstrated superior performance across all metrics for the 3 entities, achieving average *F*_1_-scores of 79.3% (SD 0.5%) for population elements, 79.3% (SD 0.5%) for intervention elements, and 74.0% (SD 0.5%) for outcome elements. The empirical findings substantiated that, when applied subsequent to prompt tuning, PEFT further enhanced the model’s overall extraction performance, with element-dependent differences across PICO categories. Specifically, intervention elements improved from 75.2% (SD 0.9%) to 79.3% (SD 0.5%), and outcome elements increased from 65.3% (SD 1.0%) to 74.0% (SD 0.5%), whereas the *F*_1_-score for population elements declined from 81.3% (SD 0.8%) with COT prompting to 79.3% (SD 0.5%) after QLoRA fine-tuning. Although the enhancement magnitude was less pronounced than in recognition tasks, these findings reaffirm the performance advantages of fine-tuned methods. This pattern suggests that the advantage of quantization is more evident in extraction tasks, whereas in the relatively simpler recognition setting, QLoRA does not fully realize its potential.

In addition to predictive performance, we further compared the computational efficiency of the 3 PEFT methods in 2 representative task settings, namely, PubMed-PICO recognition and EBM-NLP extraction.

As shown in Table 8, QLoRA consistently reduced peak GPU memory use relative to LoRA in both task settings. In the PubMed-PICO recognition task, QLoRA reduced peak GPU memory from 16.2 GB to 12.3 GB while maintaining the same inference latency. In the EBM-NLP extraction task, QLoRA likewise required less peak GPU memory than LoRA (18.0 GB vs 21.6 GB).

**Table 8.** Computational efficiency of parameter-efficient fine-tuning methods. Inference latency (ms per sample) represents the total accumulated time required to extract all relevant population, intervention, comparison, and outcome elements from a single sample.

Task and method	Training time (h)	Peak GPU^a^ memory use (GB)	Inference latency (ms per sample)	GPU-hours	Effective deployment size (GB)
PubMed-PICO recognition task					
QLoRA^b^	2.26	12.3	113	2.26	6.78
LoRA^c^	2.10	16.2	113	2.10	6.78
Freeze	1.30	19.4	113	1.30	6.5
EBM-NLP extraction task					
QLoRA	3.85	18.0	135	3.85	6.78
LoRA	3.74	21.6	135	3.74	6.78
Freeze	1.72	19.9	135	1.72	6.5

a GPU: graphics processing unit.

b QLoRA: quantized low-rank adaptation.

c LoRA: low-rank adaptation.

In the NICTA-PIBOSO recognition task, multiple research teams, including Lui [33], Amini et al [26], and Dernoncourt et al [34], conducted comparable experiments to those by Jin and Szolovits [24]. As detailed in Table 9, which summarizes *F*_1_-score comparisons from multiple studies on the NICTA-PIBOSO dataset, our model outperformed previous methods in 2 of the 3 elements (intervention and outcome). Notably, while our average population element *F*_1_-score (72.5%, SD 0.7%) was marginally lower than that reported by Jin and Szolovits [24], we achieved the best reported *F*_1_-scores for both intervention (mean 72.4%, SD 0.7%) and outcome (mean 96.6%, SD 0.2%) elements. This comprehensive comparison highlights the competitive performance of our approach across key evaluation metrics.

**Table 9.** *F*_1_-scores on the test set of the NICTA-PIBOSO dataset^a^.

	Population element (%), *F*_1_-score	Intervention element (%), *F*_1_-score	Outcome element (%), *F*_1_-score
Lui [33]	58.0	34.0	89.0
Amini et al [26]	51.0	35.0	86.0
Dernoncourt et al [34]	59.2	36.5	89.1
Jin and Szolovits [24]	*74.6* ^b^	64.3	90.9
Our model, mean (SD)	72.5 (0.7)	*72.4* (*0.7*)	*96.6* (*0.2*)

a This table follows the official NICTA-PIBOSO train-test split comparison reported in prior studies. Similarly, in the EBM-NLP extraction task, our model demonstrated superior performance compared to existing approaches. The mean *F*_1_-score across the population, intervention, and outcome elements in this study reached 77.5%, outperforming established benchmarks, including SciBERT [35] (73.1%), BioLinkBERT-Large [36] (74.1%), BioBERT [37] (73.1%), and AlpaPICO [38] (64.8%). These comparative analyses collectively demonstrate the methodological rigor of our experimental design and validate the enhanced performance of our proposed model through external benchmarking against selected strong prior baselines.

b The highest *F*_1_-score achieved for each PICO element among all compared methods.

We additionally examined several representative incorrect predictions from the 3 benchmark datasets. As shown in Table 10, the observed errors mainly fell into 4 categories: incorrect extraction, entity omission, boundary mismatch, and sentence-level category confusion.

**Table 10.** Representative population, intervention, comparison, and outcome (PICO) error cases.

Error type	Dataset and task	Original text	Gold-standard annotation	Model prediction	Explanation
Incorrect extraction	EBM-NLP; extraction	“To evaluate the efficacy and safety of two 1-week low-dose triple-therapy drug regimens involving antisecretory drugs for Helicobacter pylori infection, 99 patients with H. pylori infection were treated with either lansoprazole (LPZ) or ranitidine (RNT) used together with clarithromycin (CAM) and metrinidazole (MTZ).”	Intervention: “antisecretory drugs,” “lansoprazole,” “ranitidine,” “clarithromycin (CAM),” and “metrinidazole (MTZ)”; outcome: “efficacy and safety”; population: “99” and “H. pylori infection”	Intervention: “lansoprazole (LPZ),” “ranitidine (RNT),” “clarithromycin (CAM),” and “metrinidazole (MTZ)”; outcome: “efficacy and safety”; population: “99” and “H. pylori infection”	The prediction omitted the broader intervention phrase “antisecretory drugs.”
Entity omission	EBM-NLP; extraction	“The cure rate of H. pylori infection was 88 % in the LCM group; 95 % CI 79-97 and 92% in the RCM group; 95 % CI 84-99.”	Intervention: “LCM” and “RCM”; outcome: “cure rate of H. pylori infection”	Intervention: “RCM”; outcome: “cure rate of H. pylori infection”	The model correctly extracted the outcome but missed the “LCM” intervention, identifying only “RCM.”
Boundary mismatch	EBM-NLP; extraction	“Seventy-eight women were randomized to receive 45 mg of hyperbaric 1.5 % lidocaine with or without 10 microg of fentanyl.”	Intervention: “45 mg of hyperbaric 1.5 % lidocaine with or without 10 microg of fentanyl”; population: “Seventy-eight women”	Intervention: “lidocaine with or without fentanyl”; population: “women”	The prediction retained the core concepts but failed to match the complete gold standard annotation, especially dosage and population information.
Sentence-level category confusion	PubMed-PICO; recognition	“Referral of postsurgical CRC survivors to weekly CR exercise classes and information sessions.”	Intervention	Population	The sentence contains both population and intervention cues, whereas its dominant PICO role is “intervention.”
Sentence-level category confusion	NICTA-PIBOSO; recognition	“Complete urinary continence was achieved in 37/44 men (84.1%) after 6 months and in 43/44 patients (97.7%) 1 year after surgery.”	Outcome	Population	The sentence contains population terms within an outcome-reporting statement, leading to outcome-population confusion.

## Discussion

### Principal Findings

This study systematically investigated prompt engineering and PEFT techniques for PICO element recognition and extraction driven by the practical demands of intelligent EBM development. Focusing on PICO elements in medical literature, we propose a novel framework integrating prompt engineering and PEFT to automate PICO element processing. Our methodology involved two key phases: (1) designing prompt templates for specific tasks, evaluating the basic reasoning ability of models based on prompt engineering, and using the optimal prompt configuration; and (2) based on prompt engineering, implementing PEFT to further optimize the performance of the model.

The experiment revealed 3 significant findings. First, our divide-and-conquer approach using COT prompting achieved the strongest average performance among the prompt engineering strategies across recognition and extraction tasks, indicating enhanced reasoning capability for interpreting complex medical contexts. This superiority suggests that structured cognitive prompting can effectively improve semantic comprehension and precision in recognition tasks. One explanation for COT’s superior performance is that the relatively small-parameter models used in our experiments may struggle to process the intricate reasoning steps inherent in MPR or the implicit reasoning demonstrations typical of ICL. Consequently, these models likely derive greater benefit from the explicit, step-by-step reasoning provided by COT. Furthermore, considering the moderate complexity of the PICO recognition and extraction tasks, a sequential reasoning strategy such as COT aligns well with guiding model cognition in an explicit and systematic manner, thus improving task-specific performance. Second, in PEFT, for the recognition task, LoRA showed the best results on the PubMed-PICO dataset, and QLoRA showed the best results on the NICTA-PIBOSO dataset. For the extraction task, QLoRA performed best. These results validate PEFT’s effectiveness in computing power–constrained clinical scenarios, providing technical support for deploying lightweight models in real-world medical applications. To the best of our knowledge, this work presents the first systematic comparison between prompt engineering and further PEFT for PICO processing. This establishes clear methodological guidelines for PICO tasks: the optimized prompt engineering strategy is feasible, and the task effect can be further improved through supervision and fine-tuning when the training data are sufficient.

### Limitations

There are still some limitations to this study. First, the relatively modest performance gains in extraction tasks (eg, outcome element *F*_1_-score increasing from an average of 65.3%, SD 1.0% to 74.0%, SD 0.5%) highlight persistent challenges in entity boundary detection and long-range dependency resolution. We also observed extraction errors in cases involving ambiguous entity boundaries and sentences containing multiple relevant entities. These difficulties may be associated with the relatively small model size and the added complexity of the prompt design. For example, the prompt instruction that “only one category can be extracted from a text” may have introduced ambiguity when sentences contained multiple distinct mentions of the same PICO element type, potentially contributing to incomplete extraction. Second, as our experiments were confined to the Llama 3.2-3B architecture, whether the observed findings generalize to larger models, other model families, or medical domain–specific models remains unclear. Third, the framework has not yet been validated in real-world clinical decision support systems, so its practical utility still requires further verification. Fourth, class imbalance in NICTA-PIBOSO was not specifically investigated in this study. This should be addressed in future research. Finally, potential data contamination cannot be fully excluded for Llama 3.2-3B given that PubMed-PICO, NICTA-PIBOSO, and EBM-NLP are publicly available benchmark datasets; therefore, part of the baseline performance may reflect prior exposure rather than true zero-shot comprehension.

### Future Directions

Future research should explore hybrid strategies that combine syntactic parsing with contextual augmentation to better address the challenges in entity boundary detection and long-range dependency resolution. Promising directions also include integrating LLMs with knowledge graphs [39] to capture implicit medical logic and using advanced models (eg, DeepSeek 671B) [40] to distill smaller LLMs, thereby potentially enhancing efficiency while achieving superior performance. In addition, implementing our framework in clinical decision support systems could further validate its real-world utility and extend its applicability to population, intervention, comparison, outcome, and study design (PICOS) framework analysis.

### Conclusions

This study demonstrated that the application of advanced prompt engineering and PEFT techniques can substantially enhance the ability of LLMs to recognize and extract PICO elements from biomedical literature, achieving notable performance gains across diverse datasets. These findings underscore the potential utility of LLMs in the field of EBM and provide empirical support for future innovative endeavors in this domain.

## Supplementary material

10.2196/91215 Multimedia Appendix 1 Prompt templates used for recognition and extraction tasks.

## Abbreviations

COT: chain of thought
EBM: evidence-based medicine
GPU: graphics processing unit
ICL: in-context learning
LLM: large language model
LoRA: low-rank adaptation
MPR: multi-path reasoning
NER: named entity recognition
PEFT: parameter-efficient fine-tuning
PICO: population, intervention, comparison, and outcome
PICOS: population, intervention, comparison, outcome, and study design
QLoRA: quantized low-rank adaptation
