# Supplementary material for: Advancing Evidence-Based Medicine for Population, Intervention, Comparison, and Outcome Element Recognition and Extraction in Medical Literature: Large Language Model Approach
Source: J Med Internet Res. 2026 Aug 14;28:e91215. doi: 10.2196/91215 (PMC13475930; doi:10.2196/91215)
Supplement: Supplementary file 1 [file jmir-v28-e91215-s001.docx]

**1. Baseline prompt for recognition task**

The following is a description of a task, please return the corresponding results according to the task requirements.

Task description

$$$

Your task is to classify the following evidence-based medical literature texts, there are 3 categories, namely population-P, intervention-I, and outcome-O, and only one category can be selected for each text.

Your output needs to be in JSON format.

Instruction

$$$

In 79.1% of patients visual acuity was improved or unchanged following surgery, and in 20.9% there was a reduction of Snellen acuity.

Input

$$$

Output:

Output

**2. Baseline prompt for extraction task**

The following is a description of a task, please return the corresponding results according to the task requirements.

Task description

$$$

Your task is to extract the Intervention (the treatment or action being studied) from the following medical summary. Intervention refers to the specific treatment, procedure, or strategy applied in the study and only one category can be extracted for each text.

Your output needs to be in JSON format.

Instruction

$$$

By contrast, the GH secretion was significantly lower the nights the healthy controls were given octreotide

Input

$$$

Output:

Output

**3. ICL prompt for recognition task**

$$$

Your task is to classify the following evidence-based medical literature texts, there are 3 categories, namely population-P, intervention-I, and outcome-O, and only one category can be selected for each text.

Your output needs to be in JSON format.

Here are some examples:

Example 1:

"input": "They were randomly assigned to receive @ minutes of knee-joint cryotherapy , @ hour of therapeutic rehabilitation exercises , or cryotherapy followed by exercises .",

{"output": "I"}

Example 2:

"input": "We measured quadriceps Hoffmann reflex, normalized maximal voluntary isometric contraction torque , central activation ratio using the superimposed-burst technique , and patient-reported outcomes before and after the intervention period .",

{"output": "O"}

**4. ICL prompt for extraction task**

$$$

Your task is to extract the Intervention (the treatment or action being studied) from the following medical summary. Intervention refers to the specific treatment, procedure, or strategy applied in the study and only one category can be extracted for each text.

Your output needs to be in JSON format.

Here are a few examples:

Example 1:

input: By contrast , the GH secretion was significantly lower the nights the healthy controls were given octreotide ( GH AUC 22.6+/-5.4 mU/l x h during octreotide and 126.6+/-21.9 mU/l x h during saline ; p < 0.01 ) .

output: {"INT": "octreotide"}

Example 2:

input: No significant differences were observed in serum estrone , estradiol , sex hormone-binding globulin , dehydroepiandrosterone sulfate , prolactin , or progesterone concentrations with soy feeding in the non-OC or the OC group .

output: {"INT": "soy"}

**5. COT prompt for recognition task**

$$$

Your task is to classify the following evidence-based medical literature texts, there are 3 categories, namely population-P, intervention-I, and outcome-O, and only one category can be selected for each text.

Your output needs to be in JSON format.

To determine the category, follow these steps:

1. Analyze the text to identify its focus:

- Population (P): The text describes the group of individuals or subjects involved in the study.

- Intervention (I): The text describes the specific treatment, procedure, or strategy being applied or studied.

- Outcome (O): The text describes the results, measures, or effects observed in the study.

2. Use the analysis to reason step by step and identify the most appropriate category.

3. Output the category.

**6. COT prompt for extraction task**

$$$

Your task is to extract the Intervention (the treatment or action being studied) from the following medical summary. Intervention refers to the specific treatment, procedure, or strategy applied in the study and only one category can be extracted for each text.

Your output needs to be in JSON format.

To extract the Intervention, follow these steps:

1. Identify the treatment, procedure, or strategy mentioned in the text.

2. Check if it is explicitly described as being tested, applied, or compared in the study.

3. Output the specific Intervention in a concise format.

**7. MPR prompt for recognition task**

$$$

Your task is to classify the following evidence-based medical literature texts, there are 3 categories, namely population-P, intervention-I, and outcome-O, and only one category can be selected for each text.

Your output needs to be in JSON format.

To determine the correct category, follow these steps:

Step 1: Generate Multiple Reasoning Paths – Construct three different reasoning paths, each corresponding to a categorization hypothesis: Path One assumes the sentence mainly describes population characteristics (P); Path Two assumes it mainly describes the intervention (I); and Path Three assumes it mainly describes the outcome (O).

Step 2: Evaluate Each Path – Conduct an in-depth analysis for each path by comparing how well each aligns with the actual content of the sentence.

Step 3: Integrate Decision and Output Result – Based on the evaluation results from each path, comprehensively determine the classification that best fits the content of the sentence, and output the final classification result (P, I, or O).

**8. MPR prompt for extraction task**

$$$

Your task is to extract the Intervention (the treatment or action being studied) from the following medical summary. Intervention refers to the specific treatment, procedure, or strategy applied in the study and only one category can be extracted for each text.

Your output needs to be in JSON format.

To extract the Intervention, follow these steps, breaking them into separate branches for better analysis:

Step 1: In-depth Analysis of the Input Sentence – Carefully read the sentence, focusing on identifying the parts that describe the intervention (I);

Step 2: Constructing Multiple I Element Extraction Paths – Generate several extraction schemes for the I element, with each path based on different clues or strategies:

Path A involves using the intervention verbs or noun phrases directly described in the sentence to extract the relevant original text;

Path B captures potential implicit intervention information by analyzing modifiers and qualifiers;

Path C infers a subtler yet reasonable description of the intervention by combining contextual logic;

Step 3: Comparison and Analysis – Conduct a comprehensive comparison of each I element extraction path, evaluating how well each scheme matches the actual information in the sentence, and emphasize that if clear I element information is present, the corresponding path must be prioritized;

Step 4: Integration and Output – Integrate the analysis results from all paths to ultimately determine and output the original text fragment of the I element.
